# Alzheimer's Disease Biomarker Analysis Using Targeted Mass Spectrometry

**DOI:** 10.1016/j.mcpro.2024.100721

**Published:** 2024-01-20

**Authors:** Johan Gobom, Ann Brinkmalm, Gunnar Brinkmalm, Kaj Blennow, Henrik Zetterberg

**Affiliations:** 1 Department of Psychiatry and Neurochemistry, Institute of Neuroscience and Physiology, The Sahlgrenska Academy at the University of Gothenburg, Mölndal, Sweden; 2 Clinical Neurochemistry Laboratory, Sahlgrenska University Hospital, Mölndal, Sweden; 3 Department of Neurodegenerative Disease, UCL Institute of Neurology, London, UK; 4 UK Dementia Research Institute at UCL, London, UK; 5 Hong Kong Center for Neurodegenerative Diseases, Clear Water Bay, Hong Kong, China; 6 Wisconsin Alzheimer’s Disease Research Center, University of Wisconsin School of Medicine and Public Health, University of Wisconsin-Madison, Madison, Wisconsin, USA

**Keywords:** Alzheimer's disease, biomarkers, amyloid, tau, targeted mass spectrometry

## Abstract

Alzheimer’s disease (AD) is characterized by several neuropathological changes, mainly extracellular amyloid aggregates (plaques), intraneuronal inclusions of phosphorylated tau (tangles), as well as neuronal and synaptic degeneration, accompanied by tissue reactions to these processes (astrocytosis and microglial activation) that precede neuronal network disturbances in the symptomatic phase of the disease. A number of biomarkers for these brain tissue changes have been developed, mainly using immunoassays. In this review, we discuss how targeted mass spectrometry (TMS) can be used to validate and further characterize classes of biomarkers reflecting different AD pathologies, such as tau- and amyloid-beta pathologies, synaptic dysfunction, lysosomal dysregulation, and axonal damage, and the prospect of using TMS to measure these proteins in clinical research and diagnosis. TMS advantages and disadvantages in relation to immunoassays are discussed, and complementary aspects of the technologies are discussed.

## Highlights

• Targeted mass spectrometry (MS) plays an important role in validating and characterizing biomarkers of the different pathologies involved in Alzheimer’s disease.
• Biomarkers found in cerebrospinal fluid and blood can identify pathological alterations years before symptoms manifest, proving pivotal for early patient diagnosis and in the context of clinical trials.
• Although MS is currently mainly applied in the exploration and validation phases, advancements in high-end instrumentation geared toward enhanced robustness could facilitate broader application of MS in both clinical research and routine settings.

## Graphical Abstract

**Figure:**
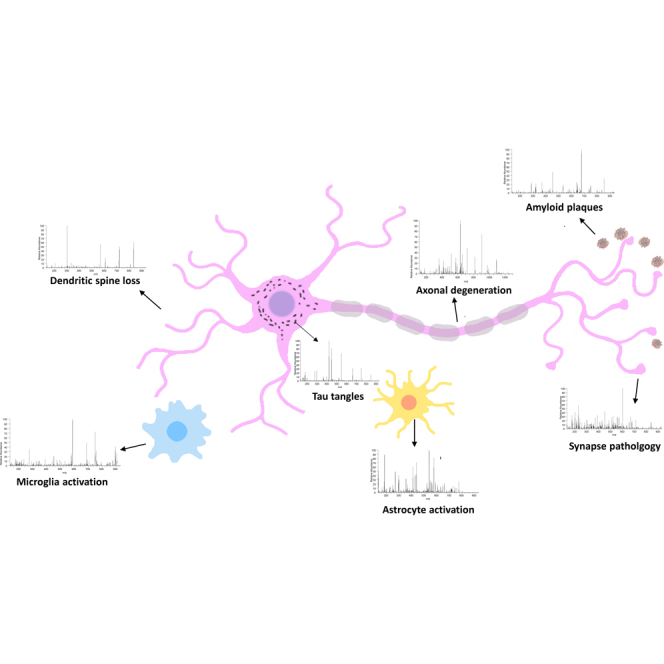

## In Brief

Alzheimer’s disease is accompanied by several neuropathological changes, including amyloid plaques, tau tangles, axonal, synaptic, and dendritic degeneration, and tissue reactions, such as astrocytosis, microglial activation, and lysosomal dysregulation. Many of the changes are reflected in cerebrospinal fluid and blood long before the onset of symptoms, and several biomarkers monitoring this have been identified. We review the use of targeted mass spectrometry to measure these biomarkers and discuss the prospect and limitations of using the technique in clinical research and diagnostics.

Alzheimer’s disease (AD) is the most common neurodegenerative disease, accounting for 50 to 70% of all neurodegenerative dementia cases, and affecting an estimated 50 million people worldwide (1). AD progresses through a continuum, beginning in a preclinical phase with initial pathological changes in the brain but without symptoms, proceeding to a prodromal stage in which mild memory and cognitive symptoms begin to manifest, and finally to AD dementia, characterized by a marked loss of short-term memory, impaired reasoning skills and ability to perform complex tasks, language impairment, and spatial disorientation (1). The onset of AD is usually above 65 years of age, but around 1% of patients have earlier onset. While early-onset AD is strongly linked to genetic mutations, such strong links have not been found for late-onset AD, although several genetic risk factors have been identified (1).

The main pathological changes in AD, described by Alois Alzheimer over a century ago, are two types of aggregates in the brain: intraneuronal amyloid plaques and extracellular neurofibrillary tangles (NFTs). The main constituents of the amyloid plaques are amyloid β (Aβ) peptides derived from the amyloid precursor protein, mainly Aβ1–42. According to the amyloid cascade hypothesis, which since 30 years is the leading theory on the etiology of AD (2), an imbalance of the activities of the three protease complexes, α-, β-, and γ-secretases, leads to decreased clearance of Aβ1–42, resulting in the formation of neurotoxic peptide oligomers and plaques. Aβ is currently the primary target for AD drug development. Treatment with therapeutic antibodies targeting Aβ peptides has recently shown promising results in clinical trials (3, 4, 5, 6). The NFTs consist mainly of truncated and abnormally phosphorylated forms of the tau protein (p-tau) (7). While amyloid plaque pathology occurs exclusively in AD, aggregation of tau is involved in many neurodegenerative diseases, which are collectively termed *tauopathies*. Besides plaques and tangles, a majority of AD patients are also affected by several other pathologies including α-synucleinopathy (Lewy bodies) and TDP-43 pathology (8). An important goal in current research on AD is to develop fluid biomarkers that can detect these pathologies as such markers could be used to monitor disease progress in patients and measure the effects of drug treatment.

**Fig. 1:**
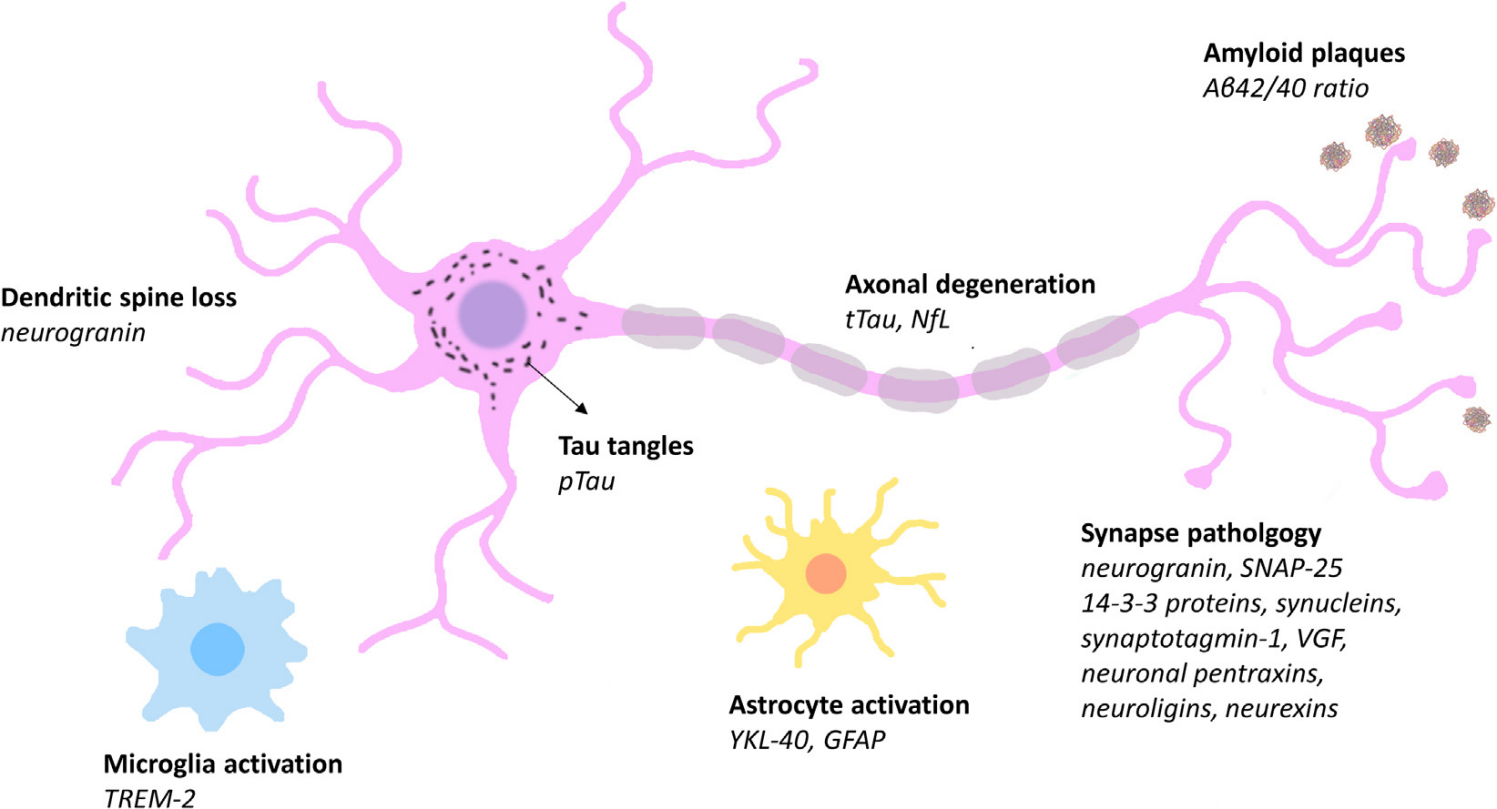
**Cellular origin of fluid biomarkers of Alzheimer’s disease (AD).** The figure illustrates the main pathological changes affecting neural cells in AD and the proteins whose release into the cerebrospinal fluid (CSF) and plasma are altered as a result. The Aβ42/40 ratio in CSF and plasma is a direct marker of amyloid pathology, the earliest observed pathological change in AD. Affected neurons secrete total and multiply phosphorylated tau (t-tau and p-tau) at an increased rate, leading to higher concentrations of both proteins in CSF and increased pTau concentration in plasma. Axonal degeneration leads to leakage of neurofilament light polypeptide (NfL) and tau into the CSF and blood. Synaptic loss as well as synaptic hyperplasticity leads to changes in a host of synapse-associated proteins, including neurogranin, SNAP-25, and synaptotagmin-1. Dendritic spine loss is closely linked to synaptic dysfunction and results in the release of neurogranin into the CSF. Glial activation is part of the neuroinflammatory response to AD pathologies, leading to the release of TREM-2 from microglia and YKL-40 and GFAP from astroglia.

In this review, we discuss how targeted mass spectrometry (TMS) is being used to validate and further characterize classes of biomarkers that reflect different pathologies involved in AD and the prospect of using TMS in clinical research and diagnosis Figure 1.

## Analytical Approaches and Instrumentation for TMS

TMS is a broad term that refers to the application of an analytical focus to a predetermined subset of compounds (9). Frequently, it means using a mass spectrometric method that only acquires data from preselected peptides (or other compounds) with the aim to quantify these, in contrast to data-dependent acquisition, which is typically used in explorative proteomics studies and aims to detect and identify as large part of the sample components as possible. The classical form of TMS, and still most widely used in clinical settings, is selected reaction monitoring (SRM), which is performed using triple–quadrupole mass spectrometers. More recently, by replacing the last quadrupole with a mass analyzer that records full fragment ion spectra (such as time of flight, Orbitrap, or linear ion trap), parallel reaction monitoring (PRM) has emerged, improving specificity and omitting the requirement to preselect transitions (10). During the last decade, non-TMS methods based on data-independent acquisition (DIA) have become increasingly used, with the development of faster and more sensitive instrumentation (11). In DIA, all ions in predefined and wide *m/z* windows are fragmented together, and identification and quantification are performed by querying data against libraries of previously recorded fragment ion and retention time data. With the newest instruments, the isolation windows have shrunk further, approaching that of SRM–PRM, which may in the future close the gap between TMS and DIA mass spectrometry (MS) (12, 13, 14).

However, the term *targeted* can also refer to the introduction of a selective purification step in the sample preparation, for example, immunoprecipitation (IP) of a particular protein. This is often required in case of low-abundant proteins in high-protein background matrices, such as plasma, serum, or cerebrospinal fluid (CSF). The mass spectrometric analysis can then be either targeted or nontargeted. In this review, both these strategies are discussed in the context of AD biomarkers.

## The Core Biomarkers

CSF Aβ1–42, total tau (t-tau), and p-tau are the core biomarkers and have been implemented in the research criteria for AD, IWG-2, and NIA-AA (15).

### Beta Amyloid

Aβ1–42 in CSF is one of the core AD biomarkers, showing a decrease by approximately 50% in patients who actively deposit Aβ in the brain (16). CSF Aβ1–42 is used extensively in clinical research and increasingly also to support clinical diagnosis of patients, often measured as the ratio of Aβ1–42/Aβ1–40, which improves performance, conceivably because the ratio accounts for individual and nonpathological variation in total CSF Aβ peptide load (17, 18, 19). While validated, fully automatic immunoassay instruments are available from several manufacturers to measure CSF Aβ1–42 and Aβ1–42/Aβ1–40 in clinical settings, including Food and Drug Administration–approved methods (20), MS is extensively used in research to address a wide range of research questions.

IP combined with MALDI-TOF MS provides a rapid means to measure multiple Aβ peptides in CSF (21, 22, 23), which has proven valuable when studying proteolytic processing of the peptides linked to disease or treatment (24, 25, 26, 27). For more sensitive and precise measurement, LC–MS is normally used. The propensity of Aβ peptides, particularly Aβ1–42, to form aggregates and adsorb to surfaces presents some problems, but these can be overcome by performing reversed-phase LC–MS at basic pH, at which the solubility of Aβ1–42 and Aβ1–40 is higher compared with acidic pH (28), enabling the development of high-precision SRM assays (29, 30). Based on these assays, a reference method procedure has been developed (31), allowing assignment of concentrations to certified reference materials, which have been used to harmonize measurements between commercial immunoassays (32). Aβ1–42 metabolism has also been studied *in vivo* by stable isotope–labeled kinetics (33), enabling precise measurement of the kinetics of Aβ peptide production and clearance. TMS has also been used to measure glycosylated Aβ peptides (34).

Aβ1–42 and other Aβ peptides can also be measured in plasma by using IP in combination with LC–MS (35, 36, 37) and MALDI–MS (38), although their biomarker performance is lower in plasma than in CSF, possibly because of peripheral origin of plasma Aβ peptides.

### Tau

Under physiological conditions, tau is involved in regulating the self-assembly of tubulin into microtubules in neurons and helps stabilizing the axonal cytoskeleton in a dynamic fashion modulated by phosphorylation. In AD, tau becomes hyperphosphorylated (p-tau), causing it to detach from the microtubules, leading to axonal degeneration and the aberrant aggregation of tau into paired helical filaments, which in turn assemble into NFTs (39). There are six splicing variants of tau that differ in the number of repeats in the microtubule-binding domain (3R or 4R) (40) as well as by the presence or the absence of N-terminal inserts (4). Different tauopathies vary in their relative abundance of 3R and 4R in the NFTs, with AD exhibiting a mixture of 3R and 4R, corticobasal syndrome (corticobasal degeneration) and progressive supranuclear palsy containing solely 4R, and Pick’s disease harboring 3R tau only (5, 6, 7). Data suggest that the ratio of splice variants determines the conformation of p-tau in the aggregates, which may affect its interactions with surrounding proteins to mediate toxic effects of p-tau and influence the development of different tauopathies (8).

In AD patients, the t-tau concentration (t-tau, including both phosphorylated and nonphosphorylated forms) in CSF increases twofold to fourfold (41, 42, 43, 44, 45). P-tau, most commonly measured by immunoassays that detect phosphorylation at amino acid Thr-181 (pT181), located in the midregion of the protein, increases by a similar fold change (42, 43, 46, 47), but while elevated CSF t-tau can also reflect acute neuroaxonal injury in Creutzfeldt–Jakob disease (CJD) (48), stroke (49), and brain trauma (50), increased p-tau is more specific to AD (43). Other phosphoepitopes, including pS199, pT217, and pT231 as well as the C-terminal residues pS396 and pS404 (51, 52, 53, 54, 55, 56, 57) appear to have different sensitivity and specificity to detect AD, prompting an interest in detailed characterization of tau phosphorylation in AD and other tauopathies to better understand their involvement in the disease process.

Using immunoassays to elucidate the role of specific phosphoepitopes and processed forms of tau in AD has proven complicated, as the measurements depend on the recognition epitopes in tau of the antibodies used, which may lead to discrimination among differently processed forms of tau. For example, while one study showed improved biomarker performance of pT217 compared with pT181 in AD (58), two studies that compared pT181 and pT217 in CSF, using the same N-terminal antibody (Tau12) as the detector in the two assays, suggested identical diagnostic performance of these two pTau variants (59, 60). The high specificity of TMS makes the technique well suited to shed light on such discrepancies. In brain, where tau is an abundant protein, characterizing tau proved straightforward, identifying post-translational modifications, such as methylation, glycosylation, ubiquitinylation, and phosphorylation at more than 80 sites (61, 62, 63), whereas in CSF, the relatively lower concentration of tau previously necessitated the use of IP for its characterization (64, 65). As in brain, further characterization of CSF tau revealed a complex pattern of fragments (66) and phosphorylation (67). A longitudinal study of patients with dominantly inherited AD found that phosphorylation at Thr-217 and Thr-181 occurred significantly earlier than at Thr-205, beginning as early as 20 years before the onset of symptoms (68).

TMS methods have been developed for use in clinical studies to evaluate the biological relevance of the identified tau fragments. The unusual property of tau of being soluble in 2.5% perchloric acid (69) made it possible to replace the costly and time-consuming IP step with a simpler protein precipitation step to remove abundant CSF proteins, enabling antibody-free quantification of tryptic tau peptides in human CSF by SRM on a triple quadrupole mass spectrometer (70). Transferring the method to a high-resolution orbitrap instrument allowed absolute quantification of tryptic peptides derived from multiple locations along the tau amino acid sequence, highlighting the higher abundance of midregion fragments (71, 72). Recently, antibody-free TMS detection of multiple phospho-tau epitopes was reported, confirming the increase in pT231, pT217 in early AD, and identifying pT205 as a marker that increases in importance later in the disease (73).

## Synapse-Associated Markers

Synapse dysfunction is an early event in the AD disease process. Synapse loss occurs earlier than neuron loss and correlates more robustly to cognitive decline than the numbers of plaques or tangles (74, 75). Synaptic transmission involves a train of connected processes, for example, neurotransmitter exocytosis, vesicle trafficking, docking, and fusion to the synaptic plasma membrane. Quantification of specific synaptic-associated proteins involved in these processes has proved to be a useful method to estimate synaptic function and density in the brain. Using this approach, substantial synaptic loss has been established in several cortical regions (76). A decade ago, studies on the correlation between cognitive decline in neurodegenerative diseases and synapse loss almost exclusively involved postmortem tissue. However, the significant role of synapses in the disease pathology and progression of both neurodegenerative and neuropsychiatric diseases has prompted a keen interest in detecting synapse-associated proteins in clinical samples from living patients, and during the last 10 years, several research groups, including our own, have been successful in quantifying synaptic proteins in CSF.

### Neurogranin

One of the first synaptic proteins targeted in an MS-based CSF assay was neurogranin (77). Neurogranin is a small calmodulin-binding protein involved in postsynaptic signaling pathways and primarily expressed in dendritic spines. In the initial studies, neurogranin was immunoaffinity purified from CSF using a set of different monoclonal antibodies and subsequently characterized and quantified by MALDI-TOF MS. The IP–MS analysis revealed that CSF contains many C-terminal neurogranin fragments with a variety of different truncations both at the C-terminal and N-terminal ends. A robust increase of CSF neurogranin in AD and mild cognitive impairment (MCI) because of AD (MCI–AD) compared with cognitively normal controls or cognitively stable MCI as well as MCI patients (78, 79) compared with controls or non-AD dementia patients (80) was found. In a later study, two intracellular enzymes were identified that can generate cleavages of neurogranin in the functionally important IQ domain and at the C-terminal end (calpain-1 and prolyl endopeptidase, respectively) (81). However, whether these different fragments have specific roles in different physiological or pathophysiological processes is still not known. Recently, IP and high-resolution MS further characterized neurogranin forms present in CSF but found no differences in the overall C-terminal fragment/total neurogranin ratios between samples from AD and control groups (82).

Drawing from the knowledge obtained in the initial IP–MS studies, antineurogranin antibodies directed at the C-terminal part of neurogranin have been used to develop immunoassays (77, 83, 84), and the initial findings have been verified in several studies (85) with neurogranin consistently showing increased levels in CSF of AD patients as compared with controls. Higher levels of CSF neurogranin have been reported in AD compared with MCI (78). In addition, CSF neurogranin levels have been found to correlate with brain atrophy but only in individuals with Aβ pathology (78). This increase appears to be specific for AD, as CSF from patients with other neurodegenerative diseases, with the exception of CJD (86), does not show such an increase (87). High levels of neurogranin in CSF have been shown to be predictive of more rapid decline of memory and executive function in MCI (88).

No significant differences have so far been reported in plasma levels of neurogranin between AD patients and controls (89). Here, it is important to remark that plasma concentrations of neurogranin do not correlate with CSF neurogranin, probably because of the contribution of peripherally expressed neurogranin peptides to blood neurogranin (83).

### SNAP-25

Another synaptic protein targeted early in an MS–based CSF assay is SNAP-25 (90). SNAP-25 is a presynaptic protein that together with vesicle-associated membrane proteins and syntaxins forms highly conserved SNARE complexes, which mediates Ca^2+^-triggered vesicle fusion during exocytosis.

In a similar manner as with neurogranin, monoclonal antibodies were initially used to affinity purify SNAP-25 from biochemically fractionated human brain homogenate and CSF (91). A combination of IP and high-resolution top–down and bottom–up MS was used to characterize and quantify SNAP-25. SNAP-25 was found to be N-terminally modified by methionine excision and acetylation in all brain protein fractions as well as in CSF. Similarly as neurogranin, full-length SNAP-25 was found in membrane-bound and membrane raft–associated brain fractions, but the soluble fractions and CSF mainly contained C-terminally truncated SNAP-25. An interesting finding was that the potential cleavage site for the generation of the longest truncated form of SNAP-25 (Ac-2-47) is located very close to the ionic zero layer at the center of the SNARE complex (92, 93). A high-resolution TMS assay was developed by which SNAP-25 was found to be significantly higher in patients with AD compared with controls in multiple independent cohorts (90). Interestingly, the longest soluble SNAP-25 form consistently provided a significantly better differentiation of patients with AD from controls compared with the shorter forms. Hence, later developed immunoassays have mainly targeted the longest (Ac-2-47) form identified in soluble CSF.

Summarizing, CSF SNAP-25 levels are altered early in AD, as shown by their increase as a function of more advanced stage of Aβ pathology. CSF SNAP-25 levels have also been found to be higher in *APOE* ε4 carriers, which likely reflects early Aβ-related AD changes.

### Synaptotagmin-1

Synaptotagmin-1 (Syt1) is a calcium sensor vesicle protein vital for fast synchronous neurotransmitter release in hippocampal neurons (94). In response to Ca^2+^ binding at elevated concentrations, Syt1 triggers the vesicle fusion, but the exact molecular mechanisms remain to be elucidated (95).

As with neurogranin and SNAP-25, Syt1 was initially affinity purified from biochemically fractionated human brain homogenate and CSF with monoclonal antibodies and characterized by MS (96). CSF Syt1 (C-terminally truncated after the first calcium-binding region) was found to have significantly increased levels in patients with AD and MCI, compared with controls (97). These findings have been corroborated in several later studies where Syt1 in a combination assay together with SNAP-25 was quantified in patients in the AD continuum and cognitive decline from other dementias (98). Syt1 has in most studies showed a less marked change than SNAP-25 in MCI and/or AD. However, indications that the decrease in CSF Syt1 levels is more pronounced than for SNAP-25, neurogranin, or GAP-43 in other neurodegenerative disorders compared with controls could suggest a different pathological pathway (98).

### Synuclein

The synucleins (αSyn, βSyn, and γSyn) are a family of evolutionary conserved members highly expressed in vertebrate nervous systems (99). Their precise functions are not known, but they appear to be involved in regulation of the synaptic vesicle pool and trafficking and other mechanisms involved in synaptic plasticity. αSyn is the most abundant protein of the intracellular aggregates found in Lewy bodies in Parkinson’s disease (PD), and dementia with Lewy bodies (DLB) and is widely viewed as a promising biomarker candidate for diagnosis and treatment effects (100). However, studies about αSyn in CSF in PD and other neurodegenerative disorders, mainly performed using immunoassays, have shown moderately decreased levels in PD and multiple system atrophy and increased levels in AD and CJD (101).

Quantitative information about βSyn and γSyn in CSF has not been available until recently when Oeckl *et al.* (102) presented a method for concurrent quantification of αSyn, βSyn, and γSyn in CSF by SRM. In this study, the three synucleins were characterized and compared in CSF in more detail. Synuclein concentrations showed a high correlation with each other in CSF, but in contrast to αSyn and γSyn, βSyn was not affected by blood contamination. CSF αSyn, βSyn, and γSyn concentrations were increased in AD and CJD but not altered in PD, PD dementia, DLB, and atypical parkinsonian syndromes.

SRM and IP–MS have also been used to observe increased concentrations of βSyn in CSF and blood of MCI and AD patients and in patients with CJD but not in other neurodegenerative diseases (frontotemporal dementia, DLB, PD, or amyotrophic lateral sclerosis). These findings suggest βSyn as a promising candidate blood marker for synaptic degeneration (103).

### Neuronal Pentraxins

Neuronal pentraxins constitute an evolutionary highly conserved family of scaffolding proteins (neuronal pentraxin 1 [NPTX1], neuronal pentraxin 2 [NPTX2], and neuronal pentraxin receptor [NPTXR]) located in the synaptic cleft and involved in synaptic plasticity by, for example, recruitment of postsynaptic receptors (104, 105).

Different members of the neuronal pentraxin family have been quantified in several SRM-based TMS assays and have commonly been found to have reduced CSF levels in AD in comparison with controls (106). Of special interest is that the levels seem to follow a constant linear decrease from controls to MCI and finally to AD (105). A similar pattern has also been found in PD (105), and the neuronal pentraxins seem to be potential monitoring biomarkers of the decline of cognitive and motor functions across neurodegenerative diseases; however, they seem to change later in the AD continuum than other synaptic biomarkers and possibly be more affected at later stages of AD (106).

It is particularly interesting to note, that unlike most other synaptic proteins studied, the neuronal pentraxins were found to have lower CSF concentration in AD, instead of higher. The neuronal pentraxins seem thus to be exempt from whatever AD-specific mechanism that induces higher synaptic protein CSF levels than normal.

### 14-3-3 Proteins

The 14-3-3 proteins are a family of seven (α, ε, η, γ, θ, ζ, and ς) highly conserved proteins that are abundantly expressed in the brain, especially during development. They are key regulators in several neurodevelopmental processes such as cortical development and synaptogenesis. Some of the 14-3-3 proteins have been found to be particularly enriched at the synaptic compartments (107), and several studies have explored their potential function in transmission and plasticity, of which they seem to be an important modulator (108), especially ζ/δ (109). 14-3-3 Proteins are since a long time established biomarkers of CJD (110), the protein family is both genetically and functionally linked to AD, and several explorative proteomics studies have suggested the 14-3-3 proteins as potential AD biomarkers (111, 112, 113).

Lately, four of the 14-3-3 proteins (ζ/δ, θ, η, and ε) were targeted in an SRM-based synaptic CSF panel assay (106). Across the studies, progressively higher concentrations of 14-3-3 ζ/δ were found in the AD continuum. Unlike the other synaptic proteins, for example, neurogranin, SNAP-25, and Syt1, the CSF 14-3-3 protein levels were also increased in other neurodegenerative diseases, which indicates that they could be general markers of neurodegeneration. All 14-3-3 proteins correlated strongly with each other, and it is possible that their differences in biomarker performance could be mostly because of analytical factors.

### Neurosecretory Protein VGF

VGF belongs to the granin family and is proteolytically processed to at least a dozen active peptides involved in, for example, synaptogenesis and memory formation (114). Several MS-based methods have demonstrated that VGF levels consistently decreased in brain tissue and CSF samples of patients with AD (115, 116, 117, 118, 119, 120, 121).

### Neuroligins–Neurexins

Neurexins (presynaptic) and neuroligins (postsynaptic) are two families of single-pass transmembrane synaptic adhesion proteins, which bind each other in the synaptic cleft, thus stabilizing the two compartments of the synaptic bouton (122). A PRM method for the simultaneous quantification of four neurexins (NRXN-1α, NRXN-1β, NRXN-2α, and NRXN-3α) and four neuroligins (Nlgn1, Nlgn2, Nlgn3, and Nlgn4) in CSF was developed and used to measure the proteins in a clinical cohort including CSF from controls, MCI, MCI-AD, AD, and a group of non-AD dementia (123). No difference in levels of NRXNs and Nlgns was found between AD (both at dementia and MCI stages) or controls or the non-AD dementia group for any of the targeted proteins. Neurexins and neurligins correlated strongly with each other, but only a weak correlation with the synaptic biomarkers, neurogranin and GAP-43, was found, possibly reflecting different pathogenic processing at the synapse.

### CSF Biomarker Panels for Synaptic Dysfunction

Several synapse-associated proteins mentioned previously have been included in SRM panels, enabling their simultaneous measurement in CSF. One of the most comprehensive panel assays includes 17 synaptic proteins (syntaxins, vesicle-associated membrane protein 2 [VAMP-2], activating protein 2 [AP-2] adaptor complex proteins, complexin-2, synucleins, rab GDP dissociation inhibitor alpha [rab GDI alpha], neuronal pentraxins, phosphatidylethanolamine-binding protein 1 [PEBP-1], and members of the 14-3-3 protein family) (124). The aim of this study and follow-up studies including other neurodegenerative diseases was to investigate if changes in CSF levels of different synapse-associated proteins are due to “general synapse loss,” synaptic activity, or other disease-specific processes. Briefly, the synaptic proteins studied appeared to belong to different disease-pattern groups with either decreased levels (*e.g.*, the pentraxins), increased levels (*e.g.*, 14-3-3) in all neurodegenerative diseases, or increased level in AD and decreased in non-AD (*e.g.*, neurogranin, synucleins, and SNAP-25).

## Other Biomarkers

### Apolipoprotein E

The strongest genetic risk factor for AD is apolipoprotein E (*APOE*), which in human has three major alleles, ε2, ε3, and ε4, having a global frequency of 8%, 78%, and 14%, respectively (125). The risk for developing AD is increased 2 to 3 times in people with one ε4 allele and more than ten times in people who are homozygous for ε4. In contrast, being homozygous for ε2 is protective and ε3 is neutral. The corresponding isoforms differ by single amino acid substitutions at positions 112 and 158 in the mature protein. These differences make ApoE well suited for analysis by TMS, but results from several clinical studies indicate that the strong disease association on the genetic level is not directly reflected on the protein level. One SRM-based study in plasma reported increased levels of total ApoE in MCI compared with controls (126), but two other plasma and CSF studies of AD and controls found no significant group differences (127, 128), and neither did a large CSF study that included AD, PD, PD dementia, and DLB (129), neither for total ApoE levels. Several studies, however, reported some genotype-dependent trends of total ApoE levels.

In summary, it appears that neither blood-derived nor CSF ApoE are useful biomarkers for AD despite the isoform-dependent differences of the ApoE levels and that these go in different directions in CSF and blood. The role of ApoE in AD should more likely be investigated as interaction with other species.

### Neurofilament Light

Neurofilament light (NFL) is a structural axon-stabilizing protein with particularly high expression in large-caliber myelinated axons. Its CSF and plasma levels increase in response to any neuronal injury process (130). Recently, IP–MS was used to characterize the NFL fragment pattern in CSF and a TMS assay to analyze a cohort of amyloid-positive and amyloid-negative individuals (131). Six tryptic peptides along the NFL protein chain were measured, and three tryptic peptides were found to be significantly increased in the amyloid-positive group. These results are in line with previous immunoassay-derived data on NFL but with the additional advantage showing that only mid-/rod-domain NFL fragments reflect neuroaxonal injury/degeneration.

NFL is a very relevant and sensitive biomarker and is measured in both CSF and blood using immunoassays. Despite its importance, there is still a lack of understanding exactly what species that are actually measured. For example, it has been shown that NFL exists as fragments in human CSF (131) and in a mouse model (132). On the other hand, data also point to NFL existing as a dimeric compound in CSF (133). To complicate matters further, it is conceivable that NFL (also) is present as oligomers of fragments. The fact that the nature of CSF or blood NFL is not known in sufficient detail is a plausible explanation for the lack of existing TMS assays published so far.

### Lysosomal Proteins

Increasing evidence indicates that lysosomal dysfunction may play a role in AD and other deurodegenerative diseases that involves protein aggregation (134). A study of CSF LAMP2 in a small and neurochemically defined AD *versus* non-AD cohort by IP–PRM (135) reported a moderate increase in AD. In a subsequent study, the method was further developed by omitting the IP step and expanding the assay to include a panel of 18 lysosomal proteins (AP2B1, APP, C9, CTSB, CTSD, CTSF, CTSL1, CTSZ, DDP7, GM2A, HEXB, LAMP1, LAMP2, LYZ, FUCA1, TNC2, TPP1, and ubiquitin) and applied to study AD and PD *versus* controls (136). The levels of several of the proteins were increased in AD compared with non-AD controls in the neurochemically defined cohort. However, in a clinically defined CSF cohort, the separation lost significance, although the AD levels were slightly higher than in controls. In yet another cohort stable MCI, prodromal AD, and AD were not significantly different, although prodromal AD exhibited slightly elevated levels compared with stable MCA and AD. In contrast, the PD group had decreased CSF levels of several proteins compared with the other groups studied. Taken together, these results may indicate that lysosomal dysfunction is likely not a prominent feature of AD but is worthwhile to investigate further in PD.

### Other Proteins of Potential Interest as AD Biomarkers

Triggering receptor expressed in myeloid cells 2 (TREM2) is a plasma membrane protein expressed almost exclusively by microglia and is of potential interest as a marker of microglial activation in AD (137). An SRM study in CSF from AD patients and controls reported slightly elevated TREM2 levels in AD (138).

The extracellular matrix proteoglycans brevican and neurocan have been studied using a PRM panel assay in two cohorts of CSF samples from patients with AD and vascular dementia and controls (139). While both CSF brevican and neurocan was lower for vascular dementia compared with both controls and AD, there were no or small differences between the control and AD group.

Monoubiquitin in CSF has been studied using SRM in a cohort consisting of controls, AD, and other neurodegenerative disorders (140, 141). The protein was found to be markedly increased in not only CJD in comparison to all other groups but also AD had higher CSF levels than both controls and non-AD cases, which had similar levels as controls.

Secernin-1 (SCRN-1) is a cytosolic protein, highly expressed in the brain. Using immunohistochemistry, SCRN-1 has been found to be present in plaque-associated dystrophic neurites (142), and a TMS study showed increased CSF SCRN-1 concentrations in AD patients compared with controls (143), and a strong correlation to CSF tau concentration.

## Multiprotein Panels

As TMS is well suited to handle multiple analytes, it has been used early on to establish panel assays that target multiple biomarkers (144). An SRM panel assay targeting 39 tryptic peptides from 30 proteins was used to analyze CSF from a cohort of controls, MCI, and AD in CSF (145), reporting CH3L1 (YKL-40) to be significantly increased in AD compared with controls. An SRM panel of 54 proteins was used to study a cohort of AD, DLB, and PD *versus* non-neurodegenerative controls (146). The study reported ten proteins increased in AD but not in DLB or PD (pro-orexin, ENPP2, LAMP1, transthyretin, ApoE, carnosine dipeptidase 1, ubiquitin, caboxypeptidase E, malate dehydrogenase, and insulin-like growth factor–binding protein 2) as well as 13 additional proteins that were elevated in both AD and DLB compared with controls, including CH3L1. The same panel was employed to analyze two cohorts of neurochemical AD *versus* non-AD, showing an increase in five proteins (malate dehydrogenase, ApoE, CH3L1, osteopontin, and cystatin C) in AD (147). A large SRM panel consisting of 320 tryptic peptides from 142 proteins was used to analyze samples from the ADNI-1 CSF cohort (148). In the study, hemoglobins A and B were significantly increased in MCI-to-AD converters, a result that should be interpreted with caution as increased hemoglobin can also be caused by blood contamination of CSF. NPTX2, neurosecretory protein VGF, and secretogranin-2 were also implied as top candidates as predictors for MCI-to-AD conversion. A study of CSF from an AD-control cohort using a PRM panel consisting of 29 peptides from 14 proteins found that CSF concentrations of neurosecretory protein VGF, secretogranin-2, and chromogranin A were lower in AD. In a follow-up study applied to a larger cohort of controls, (stable) MCI, MCI-AD, and AD, only neurosecretory protein VGF was significantly lower in AD (149), whereas many of the investigated proteins were increased in MCI-AD, but not for AD, compared with controls.

## Conclusion

In this review, we have highlighted several examples of how TMS is used to study proteins involved in AD pathology, both for the characterization target proteins by IP–MS and for simultaneous quantification of multiple proteins in clinical studies by panel assays. For protein characterization, a key strength of TMS is the ability to simultaneously identify and quantify specific sequence variants, post-translational modifications, and processed protein forms. This specificity has also been utilized to characterize epitopes of monoclonal antibodies, thereby aiding in the development of immunoassays. For development of protein biomarker assays, MS has proven to be a powerful tool; particularly for moderately abundant proteins that do not require IP for analyte enrichment, assay development is often relatively quick and easy, making it the technique of choice for evaluation of new biomarkers. The ability to measure multiple analytes in the same LC–MS analysis run saves time and sample and enables the creation of large panel assays. The high specificity of detection of MS, particularly for high-resolution mass analyzers, makes it a superior technique to measure modified and processed forms of proteins and peptides. Another area of use for TMS is as reference methods, because of the high specificity of detection afforded by precise molecular mass measurement, and the unbiased quantification, that is, counting analyte ions relative to a stable isotope–labeled standard.

Despite the advantages of TMS, most clinical biomarker studies of AD rely on immunoassays, and in hospital clinical routine laboratories, automated immunoassays remain the mainstay technology. One reason for this is lack of analytical robustness of high-end mass spectrometric instrumentation. High-resolution mass spectrometers are sensitive to contamination from sample components that may rapidly degrade performance. Similarly, nano- and cap-flow HPLC, which is often required to obtain sufficient detection sensitivity, is prone to clogging of columns and capillaries and variations in separation performance. Troubleshooting these types of problems is often time consuming, and requires specially trained personnel. This situation may change as instrument manufacturers and research laboratories are continuously making improvements to hardware, software, and sample preparation protocols, but in the near future, TMS is likely to primarily remain a research tool, for characterizing and developing new biomarkers.

## Conflict of interest

K. B. has served as a consultant and at advisory boards for Acumen, ALZPath, BioArctic, Biogen, Eisai, Julius Clinical, Lilly, Novartis, Ono Pharma, Prothena, Roche Diagnostics, and Siemens Healthineers; has served at data monitoring committees for Julius Clinical and Novartis; has given lectures, produced educational materials, and participated in educational programs for Biogen, Eisai, and Roche Diagnostics; and is a cofounder of Brain Biomarker Solutions in Gothenburg AB (BBS), which is a part of the GU Ventures Incubator Program, outside the work presented in this article. H.Z. has served at scientific advisory boards and/or as a consultant for AbbVie, Acumen, Alector, Alzinova, ALZPath, Annexon, Apellis, Artery Therapeutics, AZTherapies, CogRx, De nali, Eisai, Nervgen, Novo Nordisk, Optoceutics, Passage Bio, Pinteon Therapeutics, Prothena, Red Abbey Labs, reMYND, Roche, Samumed, Siemens Healthineers, Triplet Therapeutics, and Wave, has given lectures in symposia sponsored by Cellectricon, Fujirebio, Alzecure, Biogen, and Roche, and is a cofounder of Brain Biomarker Solutions in Gothenburg AB (BBS), which is a part of the GU Ventures Incubator Program (outside submitted work).
